# Musculoskeletal manifestations of bacterial endocarditis

**DOI:** 10.1590/S1516-31802000000500009

**Published:** 2000-09-01

**Authors:** Érika Bevilaqua Rangel, Álvaro Nagib Atallah

**Keywords:** Bacterial endocarditis, Musculoskeletal symptoms, Staphylococcal infection, Endocardite bacteriana, Sintomas músculoesqueléticos, Infecção estafilocócica

## Abstract

**CONTEXT:**

The incidence of staphylococcal infection has been increasing during the last 20 years.

**OBJECTIVE:**

Report a case of staphylococcal endocarditis preceded by musculoskeletal manifestations, which is a rare form of clinical presentation.

**DESIGN:**

Case report.

**CASE REPORT:**

A 45-year-old-man, without addictions and without known previous cardiopathy, was diagnosed as having definitive acute bacterial endocarditis due to Staphylococcus aureus. Its etiology was community-acquired, arising from a non-apparent primary focus. In addition, the musculoskeletal symptoms preceded the infective endocarditis (IE) by about 1 month, which occurred together with other symptoms, e.g. mycotic aneurysms and petechiae. Later, the patient showed perforation of the mitral valve and moderate mitral insufficiency with clinical control.

## RESUMO

**CONTEXTO:**

A incidência da Infecção estafilocócica vem aumentando durante os últimos 20 anos.

**OBJETIVO:**

Relatar um caso de infecção estafilocócica precedida por sintomas músculo-esqueléticos, o que é uma forma rara de apresentação clínica.

**TIPO DE ESTUDO:**

Relado de caso.

**RELATO DO CASO:**

Paciente de 45 anos, sexo masculino, sem comorbidades, não usuário de drogas endovenosas e com diagnóstico de endocardite bacteriana estafilocócica em válvula mitral, adquirida na comunidade e sem foco primário aparente. O diagnóstico foi precedido por dor lombar em cerca de 20 dias e complicada com eventos vasculares e petéquias, além de insuficiência mitral abordada apenas clinicamente.

## INTRODUCTION

There has been a growing trend for the number of both community-acquired and hospital-acquired staphylococcal infections to increase over the past 20 years. The incidence of staphylococcal endocarditis accounts for 25-35 percent of cases and is characterized by a rapid onset, high fever, frequent involvement of normal cardiac valves, and the absence of physical stigmata of the disease on initial presentation. In patients without addictions, the endocarditis is often left-sided and in 50% of cases there are embolic and neurological complications.^1^

## CASE REPORT

A 45-year-old man was admitted to the hospital because of daily fever (38 to 40°C) for 20 days, chills and lumbar pain, which worsened with movements. He had no history of smoking, alcohol abuse, recent travel, promiscuity, blood transfusion or endovenous drug addiction. There was no history of previous cardiopathy or skin lesions.

The patient appeared acutely ill. The temperature was 38°C, the pulse was 116, and the respiration was 24. The blood pressure was 120/70 mm Hg. The results of a physical examination were normal, except for a lumbar pain and tenderness. Laboratory tests showed hematocrit 33%, erythrocyte sedimentation rate 58 mm/hr, white cell count 17100/mm^3^ with normal differential count, platelet count 317000/mm^3^, creatinine 1.6 mg/dl, sodium 130 mEq/l, potassium 4.7 mEq/l, total bilirubin 0.5 mg/dl, glucose 110 mg/dl, aspartate aminotransferase 17 U/l, alanine aminotransferase 38 U/l, alkaline phosphatase 361 U/l, gamma-glutamyl-transferase 247 U/l. The urine was normal. Four specimens of blood were obtained for culture. A urine culture was sterile. Ceftriaxone was given intravenously (2 g daily). Serological tests for B and C hepatitis and for AIDS were negative. An electrocardiogram revealed sinusal tachycardia. Thoracic radiography and abdominal ultrasonography examinations were normal. Lumbar radiography showed a lytic lesion in the L4 vertebral body. A magnetic resonance imaging scan of the lumbar spine disclosed mild protrusion of the intervertebral disc between L4 and L5.

The fever persisted and on the third hospital day, the patient showed acute mental confusion, right hemiparesis, meningeal signs and hypotension (80/40 mmHg). A computed tomography scan of the brain was normal and the liquor showed 120 cells with 80 neutrophils and 8 lymphocytes. On the eighth hospital day, a grade 2 systolic murmur was present in the mitral area. Cardiac ultrasonography examination, including a transesophageal study, revealed a left ventricular ejection fraction of 83% and mitral vegetation of 12 mm with moderate mitral regurgitation. There were also petechiae in the feet. The blood culture yielded *Staphylococcus aureus* (3 of the 4 specimens obtained over a period longer than 12 hours). Oxacillin (12 g daily) and Amikacin (1.5 g daily) were started. A magnetic resonance scan of the brain revealed mycotic aneurysms in the area of the left inner capsule. The patient's condition improved considerably. On the 19th hospital day, a cardiac ultrasonography examination showed a decrease in mitral vegetation size to 10 mm with a perforation of 5 mm close to it. Digitalis was started. The vegetation completely disappeared on the 32^nd^ hospital day and a moderate mitral insufficiency developed. The patient was discharged from the hospital 6 weeks later.

## DISCUSSION

The diagnosis of definitive infective endocarditis (IE) was based on the criteria of Durack et al in Duke University.^2^ Thus, two major criteria were included, i.e. positive blood cultures and endocardiac involvement, and two minor ones, i.e. fever greater than 38°C and vascular events. Meningitis was also present.

According to Bayer, et al.^3^, 26% of infective endocarditis does not show previous valve lesions nor a history of drug addiction, as seen in the present case. And 77% of patients with a diagnosis of definitive infective endocarditis had bacteremia as a major criterion and 57% of these had 2 major criteria and 43% had 1 major criterion and 3 minor criteria. Furthermore, 57% of cases of definitive IE showed vegetation and 82% of those had 2 major criteria and 18% had 1 major criterion and 3 minor criteria. On the other hand, the vegetation could only be seen by transesophageal cardiac ultrasonography examination in 41% of the patients with definitive infective endocarditis and with the presence of vegetation.^3^

In the same way, Heiro, et al. reported that the vegetation was a major criteria in 72% of infective endocarditis cases.^4^ Moreover, the most common etiology was *Staphylococcus aureus* (23%) and *Streptococcus viridans* (17%) in a group of patients without addictions. These authors stated that there had been a temporal trend for the etiology of infective endocarditis to change over the past 30 years, and thus staphylococcal infection had increased from about 15% to 30%,^4,5^ although there was a bias insofar as the cases were reported more commonly in referral hospitals than in community ones. Also, some patients died before diagnosis due the severity of the case.^5^

The sensitivity of transthoracic cardiac ultra-sonography examination is 50 to 60% and transesophageal about 90%. The latter is more sensitive for the diagnosis of vegetation smaller than 5 mm and for prosthetic valves.^5^ Vegetation bigger than 1 to 2 cm in the left valve is related to complications, e.g. cardiac insufficiency and embolic events.^5^ And 50% of the patients with mitral regurgitation can develop congestive cardiac insufficiency.^6^ The former and latter events happened in the present case.

According to Willcox, community-acquired bacteremia due to *S. aureus* comprised 40% of the bacteremia and tended to be more severe than when hospital-acquired.^7^ Moreover, 58% of the patients with bacteremia but without addictions did not have an apparent primary focus, and 17% of these developed infective endocarditis with 68% mortality. The risk of infective endocarditis after *S. aureus* bacteremia can range from 5 to 60% and the mortality can reach 70%.^7^ To sum up, roughly 20% of patients with community-acquired *S. aureus* bacteremia and without a clinical diagnosis of infective endocarditis have presented hidden valvular vegetation or previous valvular lesions.^5^

Regarding the musculoskeletal manifestations in infective endocarditis, the lumbar pain usually occurs in subacute infective endocarditis and is secondary to either the embolization or direct involvement of the disc space with septic necrosis. Besides, it is advisable to look for a non-cardiac focus for an infection during or after the treatment in attempt to avoid the occurrence of bacteremia later, i.e. osteomyelitis, septic arthritis or paraspinal abscess.^8^ Thus, it seems that the present case showed an embolization of the intervertebral disc with subsequent bacteremia.

Churchill et al reported on 192 patients with a diagnosis of bacterial infective endocarditis, 29 (15%) of whom had only musculoskeletal involvement as the first symptom of IE and their symptoms included: arthralgia (38%), arthritis (31%), lumbar pain (23%), diffuse muscle pain (19%) and disc infection (6%). On the other hand, 84 patients (44%) also had musculoskeletal involvement during the course of the disease and in 52 of these (62%) the involvement was present among other symptoms at the diagnosis and included: arthralgia (35%), lumbar pain (29%), diffuse muscle pain (23%), local muscle pain (11.5%) and disc infection (8%).^9^

These authors also described how the diagnosis of infective endocarditis was preceded by lumbar pain for about 1.5 months, ranging from 1 to 4 months and by disc infection for about 4 months, ranging from 1.5 to 8 months.^9^ The present case had a period of less than one month before the diagnosis of infective endocarditis. However, the most common infection was streptococcal (65%) rather than staphylococcal (15.5%).

All in all, the frequency of embolization is time dependent,^5,10^ i.e. it decreases from 17 events/1000 patients/year during the first week of adequate treatment to less than 5 events/1000 patients/year during the second and third weeks of treatment.^10^

Finally, Vuielle, et al.^10^ described how roughly 70% of the vegetation does not decrease in size after adequate treatment and 12% may even increase due to fibrin and platelet deposits, valvular aneurysm or perivalvular abscess. Conversely, the decrease or resolution of the vegetation can correspond to an embolic event or healing. The latter happened with present case. And the definitive structural valvular lesions, as shown by the mitral insufficiency, were related to cardiac insufficiency and thus sometimes valvular replacement could be necessary.
